# Time to Switch to 'Rule of Three-Quarters' from 'Rule of Halves' in Hypertension? A Descriptive Study from Dakshina Kannada, South India

**DOI:** 10.7759/cureus.13142

**Published:** 2021-02-04

**Authors:** Imaad Mohammed Ismail, Subhashree Nandy, Shubhankar Adhikari, Revathi TM, Dhruv Gupta, Deeptha M, Awnish Ranjan, Aslaha Aboobacker

**Affiliations:** 1 Community Medicine, Yenepoya Medical College, Mangaluru, IND

**Keywords:** rule of halves, hypertension, prevalence, aware, treatment, adequate control, rule of three-quarters

## Abstract

Background

‘Rule of halves’ depicts the overall picture of hypertension that prevails in a community. This study was taken up to understand if the traditional ‘rule of halves’ of hypertension still prevails or is it time to shift to the proposed ‘rule of three-quarters’. The objectives of the study were: to estimate (i) the prevalence of hypertension among adult residents of Madani Nagar rural community in the Dakshina Kannada district of Karnataka state in southern India, (ii) the proportion who are aware of their disease among hypertensive patients, (iii) the proportion who are on treatment among those who are aware of their disease, and (iv) the proportion having adequate blood pressure control among those on treatment.

Materials and methods

This was a cross-sectional study carried out in Madani Nagar, Dakshina Kannada from February to April 2020. Complete enumeration method was followed and all adults aged ≥ 18 years who consented were included in the study. Data collection was done using a pre-validated questionnaire and blood pressure was recorded as per World Health Organization guidelines.

Results

A total of 661 individuals were enrolled in the study. The prevalence of hypertension was 29.2%. Only 55.4% of hypertensive patients were aware that they had the disease, and among them, 96.3% were on treatment. Among those on treatment, 58.3% had adequate control of blood pressure.

Conclusion

The hypertension pattern of the community leans more towards the traditional ‘rule of halves’ of hypertension and it is still a long way ahead until the proposed ‘rule of three-quarters’ can be achieved.

## Introduction

Hypertension is an important present-day non-communicable disease that affects more than a billion people globally, with the majority being from the low- and middle-income countries [1]. Hypertension is defined as systolic blood pressure (SBP) ≥ 140 millimetre of mercury (mm Hg) and/or diastolic blood pressure (DBP) ≥ 90 mm Hg when measured following the standard guidelines [2]. For people aged 60 and above, SBP ≥ 150 mm Hg is considered instead of 140 mm Hg.

Hypertension is known to have adverse consequences on most organs, including the heart, brain, and kidneys [1,3]. It is also one of the major causes of premature death all over the world [1]. In India, the National Family Health Survey - 4 (NFHS-4) data states that 11% of women and 15% men in the age group 15-49 years have hypertension [4]. Very often, we see that people with hypertension have no obvious signs and symptoms and therefore go undiagnosed, making it an “iceberg disease” [5].

Hypertension is known to follow the ‘rule of halves’, a concept coined by Wilber and Barrow in 1972 [6,7]. It states that merely half the hypertensive people in the general population are aware of their hypertension status, merely half of those aware of their status are being treated, and only about half of those treated have adequate control of blood pressure [7,8]. ‘Rule of halves’ of hypertension is a way to know the general picture of hypertension in any given population keeping in mind that it is a “silent disease” [9].

It has been about 50 years since the ‘rule of halves’ was initially proposed. The field of medicine has advanced during this time period. In the same time frame, the Indian medical system has similarly undergone changes, and through the implementation of the National Health Mission, India has brought about improvements in health care delivery in both urban and rural areas [10]. These measures, along with the information technology revolution that India has witnessed over the past decade, have contributed to the increase in awareness of various diseases [11]. With increased awareness, a larger proportion of the population is predisposed towards early diagnosis and treatment [12].

With passage of time and improved health care system, does the ‘rule of halves’ of hypertension still hold good? It is intriguing to know where we stand and if assumptions and theories proven years ago are valid even today. If indeed things have improved, then it may be time to propose a ‘rule of three-quarters’, where about 75% of the hypertensive individuals are aware of their disease, among them about 75% are on treatment, and among those on treatment about 75% are adequately treated. A new paradigm may be seen if the evidence is put forth by research studies in this regard.

The Dakshina Kannada district of Karnataka is a well-performing district with higher than national average literacy rate and health care facilities [13]. Madani Nagar is a rural area of the district, and there are two medical colleges situated in close proximity. We wanted to assess whether in a population like Madani Nagar, which is located in a better performing district of India, the concept of ‘rule of halves’ has shifted towards ‘rule of three-quarters’.

Therefore, this study was conducted with the following objectives: to estimate (i) the prevalence of hypertension among adult residents of Madani Nagar, (ii) the proportion who are aware of their disease among the identified hypertensive individuals, (iii) the proportion who are on treatment among those who are aware of their disease, and (iv) the proportion having adequate blood pressure control among those on treatment.

## Materials and methods

Dakshina Kannada district is situated in the coastal belt of Karnataka, India, and Mangaluru city is its headquarters [12]. Madani Nagar is a rural area having a population of 1,921, which comes under Munnuru Panchayath of the Dakshina Kannada district. This community-based cross-sectional study was conducted in Madani Nagar between February and April 2020.

Based on the national demographics estimates, we considered that the adult population constituted 60% of the total population. Hence, the total number of adult residents in the study area was estimated as 1,153 [14]. The estimated prevalence of hypertension among adults was considered as 18.3% based on a previous study on hypertension in the same district [15].

Therefore, the expected number of people with hypertension in the study area was 211. Complete enumeration method of data collection was employed and all residents who were 18 years and above were considered for the study. Pregnant women, individuals who did not consent to participate, and individuals who were not available at home even after two attempts were excluded from the study.

Ethical approval was obtained from the Institutional Ethics Committee 2 (YEC2/417) of our institution. All participants were given a participant information sheet and written informed consent was taken in the local language.

Data was collected by trained second-year undergraduate medical students under the supervision of the principal investigator using a semi-structured questionnaire through the Epicollect 5 application (Version 3.0.3, Imperial College, London). The questionnaire was validated by three subject experts. The Joint National Committee eight (JNC-8) criteria were used for defining hypertension. People were considered to have hypertension if their blood pressure was ≥ 140/90 mm Hg for adults aged below 60 years, and ≥ 150/90 mm Hg for adults aged 60 years and above [2]. People who were on anti-hypertensive drugs were also considered as having hypertension. People were considered as being aware of their hypertension status if they reported that they had been diagnosed as having hypertension by a health care professional before data collection. People with hypertension were considered to be on treatment if they were taking their medications during the period of data collection. People with hypertension who were on treatment were said to have adequate control when their SBP < 140 mm Hg and DBP < 90 mm Hg at the time of data collection. For people aged ≥ 60 years, adequate control was when their SBP < 150 mm Hg and DBP < 90 mm Hg.

To measure the blood pressure, we used a digital automatic blood pressure monitor (model Omron HEM-7130-L), which has an accuracy of +/- 3mm Hg [16]. World Health Organization's guide to physical measurements was followed for patient preparation and blood pressure recording [17]. After fifteen minutes of rest, two blood pressure readings were taken 10 minutes apart and the average reading was considered for classifying as ‘hypertension’ or ‘without hypertension’.

Data was analyzed using Microsoft Excel (version Office 2019, Microsoft Corporation, Redmond, Washington, USA) and SPSS (version 23.0, IBM Corp., Armonk, New York, USA). Continuous variables are reported in terms of mean with standard deviation (SD). Categorical variables are reported in terms of frequency and proportions. Chi-square test was used to assess association across different groups and p-value < 0.05 was considered as statistically significant.

## Results

A total of 661 individuals from the study area participated in the study. Their mean age (±SD) was 40.8 (±15.6) years. Table 1 presents the socio-demographic profile of hypertensive and normotensive individuals. Majority of the individuals (43.1%) enrolled were in the age group of 25-44 years. An increasing trend of prevalence of hypertension was observed from age 18 to 79 years, and this was found to be statistically significant (χ^2^ = 153.76, p < 0.001). About two-thirds of the study participants were females. It was observed that males had a higher prevalence of hypertension as compared to females (χ^2^ = 4.57, p = 0.03).

**Table 1:** Socio-demographic characteristics and blood pressure profiles of the study participants (N=661) PUC: Pre-University College

Variables		Total (N=661)	Hypertensive individuals (N=193)		Normotensive individuals (N=468)		χ^2^ value	p-value
			n	%	n	%		
Age	18-24	117	07	(5.9)	110	(94.1)	153.76	<0.001
	25-44	287	42	(14.6)	245	(85.4)		
	45-64	196	105	(53.6)	91	(46.4)		
	≥ 65	61	39	(63.9)	22	(36.1)		
Sex	Male	243	83	(34.2)	160	(65.8)	4.57	0.03
	Female	418	110	(26.3)	308	(73.7)		
Education	Illiterate	84	50	(59.5)	34	(40.5)	68.70	<0.001
	Primary school	51	21	(41.2)	30	(58.8)		
	Middle school	141	48	(34.0)	93	(66.0)		
	High school	170	41	(24.1)	129	(75.9)		
	PUC/post high school diploma	128	13	(10.2)	125	(97.8)		
	Graduate or postgraduate	87	20	(23.0)	67	(77.0)		

**Figure 1:**
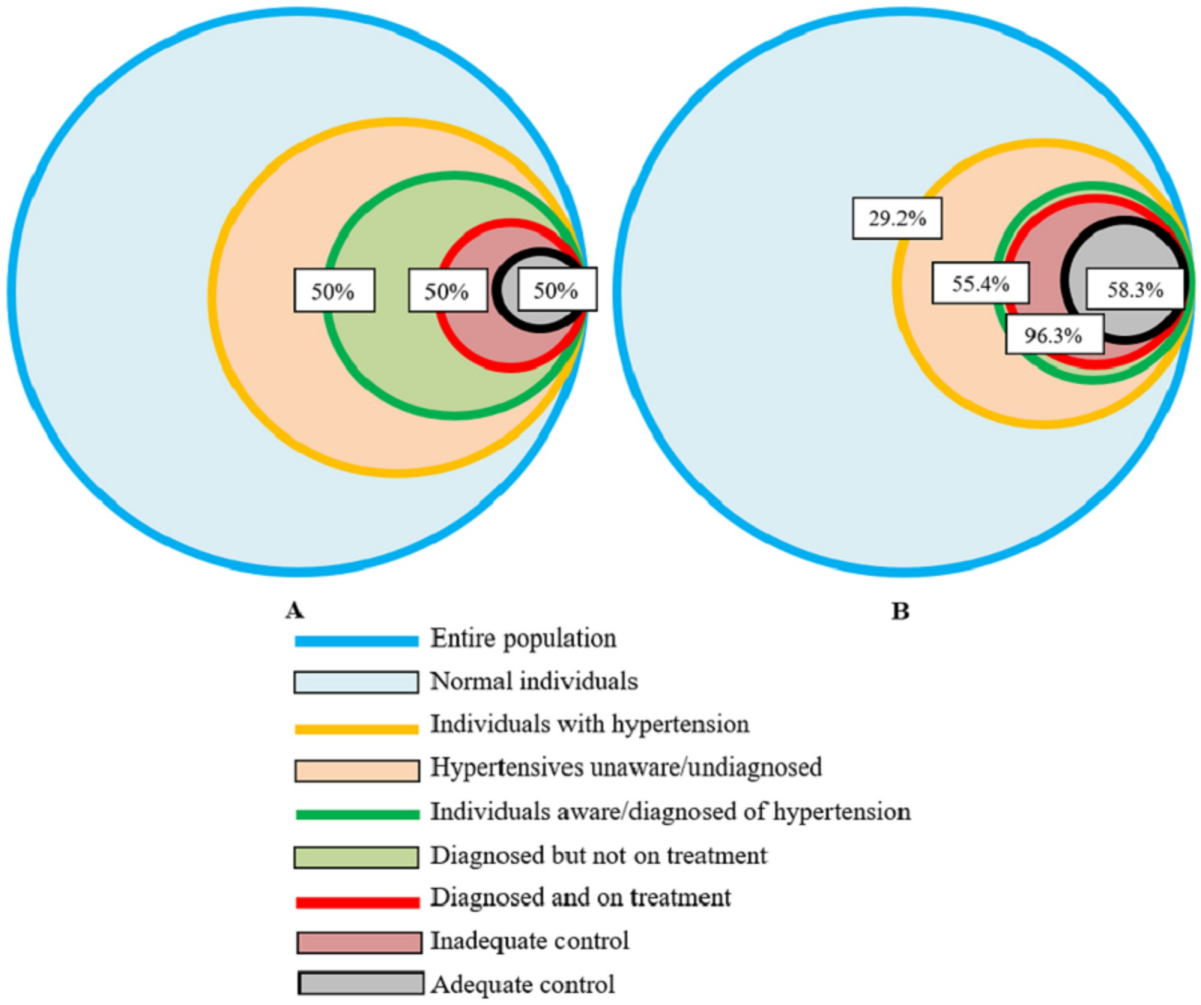
Originally depicted rule of halves of hypertension (A) and depiction of rule of halves in the study population (B)

The prevalence of hypertension in the study population was 29.9% (193/661). Out of the 193 hypertensive patients, 85 were newly diagnosed. The mean (±SD) SBP of the study participants was 129.9 (±23.1) mm Hg and the mean (±SD) DBP was 81.3 (±13.2) mm Hg. The results pertaining to the ‘rule of halves’ have been shown in Table 2. The original ‘rule of halves’ of hypertension and the depiction of the ‘rule of halves’ in the present study are presented in Figure 1. The treatment modality that was majorly followed by the hypertensive individuals was medications as prescribed by the treating physician (Table 3). Regular medication intake was noted in 85% of the hypertensive individuals with adequate blood pressure control as compared to 67.4% in hypertensive individuals without adequate blood pressure control (χ^2^ = 4.452, p = 0.035).

**Table 2:** Proportion of different categories as per rule of halves in the study population

Categories	Proportions as per rule of halves	Proportions among hypertensive individuals (n=193)
Proportion aware	55.4 (107/193)	55.4 (107/193)
Proportion on treatment	96.3 (103/107)	53.4 (103/193)
Proportion with adequate blood pressure control	58.3 (60/103)	31.1 (60/193)

**Table 3:** Management modalities adopted by hypertensive individuals (n=103)

Management modalities	n	(%)
Medications only	66	64.1
Medications + Diet	22	21.4
Medications + Physical activity	09	8.7
Medications + Diet + Physical activity	06	5.8

## Discussion

The current study looks at the hypertension prevalence, awareness, treatment and its adequacy through the lens of ‘rule of halves’ in Madani Nagar rural community of the Dakshina Kannada District of Karnataka.

A total of 661 participants were enrolled in the study. The prevalence of hypertension among the study population was 29.2%. Among the hypertensive persons, 55.4% were aware that they had the disease. Among the hypertensive people who were aware of their disease, 96.3% were taking treatment for it, and among those taking treatment, 58.3% had adequate blood pressure control.

The study sample had a good representation of different age groups with maximum representation coming from the age group of 25 to 44 years. Age is an established non-modifiable risk factor of hypertension and a similar finding was noted in the present study where the prevalence of hypertension increased with advancing age [18-20].

The total number of men and women enrolled in the study were 243 and 418, respectively. A greater number of women were enrolled in the study as the data collection was done between 9 am to 12 pm, which coincided with the working hours, and similar to other communities of India, this community has a male-dominant workforce. The prevalence of hypertension was higher in men (34.2%) than in women (26.3%). A study done by Ismail et al. also found a higher prevalence in males (22.3%) compared to females (20.1%). Although males had a higher prevalence of hypertension overall, it was observed that women had a higher prevalence (59.1%) compared to men (48.2%) in the 45-64 years age category [15]. This could be attributed to the decrease in the protective effect of the female sex hormones post-menopause [21].

When we looked at the education status and hypertension, it was observed that among the illiterate people, the prevalence of hypertension was very high (59.5%). This could be accounted by the fact that many of these illiterate people are older individuals as the literacy rate of India was only 43.6% in 1981 as compared to the current rate of 74.0% [14,22]. Multiple logistic regression analysis using age, sex and education as independent variables and hypertension as dependent variable showed that only age was significantly related to hypertension (odds ratio = 3.64, 95% confidence interval 2.69-4.93, p-value < 0.05) and not education status (odds ratio = 0.96, 95% confidence interval 0.82-1.11, p-value 0.56).

Moving on to the ‘rule of halves’ of hypertension, the study population had hypertension prevalence of 29.2%. According to the NFHS-4 data, 11% of women and 15% of men had hypertension [4]. In Dakshina Kannada, 30.1% of the women and 29.9% of the men were reported to have hypertension as per the NFHS-5 [23]. Other studies conducted in India revealed that the prevalence of hypertension in rural India ranges from 10.6% to 50.3% [18,24-26]. The methodology chosen by different authors of these studies also strongly influenced the prevalence rates reported by them, and one such important variable is the age of the population that was selected, where a higher age cut-off will have a higher prevalence rate of hypertension. The present study prevalence of 29.2% is indeed alarming as the study had a lower age cut-off of 18 years.

Hypertension is a disease that exhibits an iceberg phenomenon due to its asymptomatic nature and the mere existence of the ‘rule of halves’ reflects poorly on the awareness regarding the disease. The ‘rule of halves’ has three components. The first component talks about the proportion of hypertensive individuals that are aware of their disease status. In the present study, 107 out of 193 (55.4%) hypertensive individuals were aware that they had hypertension. The result leans towards the ‘rule of halves’ rather than the proposed ‘rule of three-quarters’, and it suggests that the secondary level of prevention (i.e., early diagnosis and treatment) has failed for about half the hypertensive individuals. A study done by Kantha et al. in a rural setting showed a similar result where 58.5% of hypertensive individuals were aware about their status of hypertension [27]. On the contrary, a study done by Hadaye et al. in the urban parts of Mumbai found that 74.4% of hypertensive individuals knew that they had hypertension [19]. Table 4 shows the comparison of ‘rule of halves’ of hypertension of the present study with studies carried out in other parts of the country.

**Table 4:** Comparison of rule of halves of hypertension of present study with studies reported from other places in India

Rule of halves	Current study (Madani Nagar, >=18 years, Rural)	Kantha K et al. (Nellore, 20-60 years, Rural) [27]	Hadaye R et al. (Mumbai, >35 years, Urban) [19]	Rao BAV et al. (Davangere, >=30 years, Urban) [29]
Prevalence of hypertension	29.2 (193/661)	41.5 (106/250)	37.5 (250/667)	36.7 (367/1000)
Proportion aware	55.4 (107/193)	58.5 (62/108)	74.4 (186/250)	34.6 (127/367)
Proportion on treatment	96.3 (103/107)	67.7 (42/62)	95.2 (177/186)	68.5 (87/127)
Proportion with adequate BP control	58.3 (60/103)	73.8 (31/42)	31.1 (55/177)	24.1 (21/87)

The finding that only about half of the hypertensive individuals were actually aware that they have hypertension is concerning, and established active interventions for early diagnosis and treatment of hypertension such as health education, annual health check-ups, opportunistic screening for hypertension in the Primary Health Centers and hypertension screening camps at the village level are required [3,28].

The second component of the ‘rule of halves’ focuses on the proportion of hypertensive individuals on treatment from among those who are aware of their disease. A striking result of 96.3% of hypertensive individuals on treatment inclined more towards the ‘rule of three-quarters’ proposed in this study than the traditional ‘rule of halves’. A study by Hadaye et al. portrays a similar result in this aspect with the proportion being 95.2% [19]. Other studies by Kantha et al. and Rao et al. show lower proportions of 67.7% and 68.5% respectively [27,29]. The present study finding suggests that once people come to know that they have hypertension and have access to a healthcare facility, then most of them will seek treatment.

The third component of the ‘rule of halves’ depicts the proportion of hypertensive persons who are on treatment that have adequate control over their blood pressure levels. As per the results of the current study, about 60% of the people taking treatment had adequate control and this leans more towards the ‘rule of halves’ than the proposed ‘rule of three-quarters’. It is also important to note that if we consider all the hypertensive individuals irrespective of whether they are on treatment or not, then a meagre 60 out of 193 (31.1%) have actually achieved adequate blood pressure control. Gupta et al. in their study reported that only 27 out of 194 (13.9%) people with hypertension had their blood pressure under control [30]. Other studies conducted by Kantha et al. and Rao et al. found that the proportion of hypertensive individuals with adequate blood pressure control was as low as 7.6% and 5.7% respectively [27,29].

Irregular medication and sole dependence on medications without any adjunct lifestyle modification in the form of dietary changes and physical activity are two important reasons for poor control of blood pressure in the study population. In view of these findings, it is recommended that healthcare providers should especially advise their hypertensive patients on regular medication and lifestyle modification. Further studies may be taken up to explore and understand the reasons for inadequate blood pressure control among the hypertensive people who are taking treatment. It is evident from the study that it is not enough if a person is on treatment, it is equally important to ensure that the treatment is successful in controlling the blood pressure.

The overall findings reveal that the study population parameters such as ‘the proportion of the hypertensive individuals who are aware that they have the disease’ and ‘the proportion of people taking treatment who have adequate blood pressure control’ leans more towards the traditional ‘rule of halves’. However, the study population parameter of ‘the proportion of hypertensive people who are aware of their disease that are taking treatment’ leans more towards the proposed ‘rule of three-quarters’. The authors of the study wanted to know if factors related to diagnosis and treatment of hypertension have sufficiently improved and if it is time to shift and adopt the proposed ‘rule of three-quarters’. The findings of the study suggest that at present the ‘rule of halves’ continues to be a better fit as compared to the ‘rule of three-quarters’.

A limitation of the current study is that the study population was derived from a village, and therefore, the results cannot be generalized. Another limitation is that data was collected in the morning hours due to which people who would have gone out for work at that time could not be enrolled in the study.

## Conclusions

A total of 661 adult residents of Madani Nagar rural community of the Dakshina Kannada District were enrolled for the study. The prevalence of hypertension was 29.2%. The prevalence rates of hypertension increased with advancing age and it was also noted to be higher among men. Only 55.4% of the hypertensive individuals were aware that they had the disease. Among the hypertensive people who were aware about their disease, 96.3% were taking treatment for it and among those taking treatment, 58.3% had adequate blood pressure control. The overall picture of hypertension in the study population leans more towards the traditional ‘rule of halves’ as of now and it is a long way ahead till the proposed ‘rule of three-quarters’ can be achieved.
